# Hydrogen-Disordering Transformation and High-Temperature and High-Pressure Phase Diagram of Brucite: Insights from Raman Spectroscopy and Electrical Conductivity

**DOI:** 10.3390/molecules31101631

**Published:** 2026-05-12

**Authors:** Mingyu Wu, Lidong Dai, Haiying Hu, Chuang Li

**Affiliations:** 1 Key Laboratory of High-Temperature and High-Pressure Study of the Earth’s Interior, Institute of Geochemistry, Chinese Academy of Sciences, Guiyang 550081, China; wumingyu@mail.gyig.ac.cn (M.W.);; 2 University of Chinese Academy of Sciences, Beijing 100049, China; 3 School of Physics and Electronic Science, Guizhou Normal University, Guiyang 550025, China

**Keywords:** brucite, Raman spectroscopy, electrical conductivity, phase transition, phase diagram

## Abstract

The structural and electrical transport properties of brucite [Mg(OH)_2_] were investigated by virtue of in situ Raman spectroscopy and alternating-current impedance spectroscopy under conditions of 0.5–20.2 GPa, 298–873 K, and different hydrostatic environments using a diamond anvil cell (DAC). Under the non-hydrostatic condition, the emergence of new Raman peaks and discontinuities in Raman shifts, FWHMs, as well as electrical conductivity well disclosed a hydrogen-disordering structural phase transition in brucite from the ordered ($P\bar{3}m1$ symmetry)–disordered ($P\bar{3}$ symmetry) structure at 5.7 GPa. Under hydrostatic condition, this transformation occurs at a lower pressure of 3.6 GPa using the 4:1 methanol–ethanol mixture (ME) as the pressure-transmitting medium (PTM), which can be attributed to the influence of deviatoric stress within the sample chamber. The reversibility of this transformation is confirmed by the recovery of Raman peaks and electrical conductivity upon decompression. Furthermore, the high-temperature and high-pressure electrical conductivity results clearly revealed a negative Clapeyron slope for the hydrogen-disordering transformation in brucite, and the corresponding high *P*–*T* phase diagram was established for the first time at pressures up to 7.0 GPa and temperatures up to 873 K, which can be expressed as P (GPa) = 8.664 (±1.511) − 0.008 (±0.002) T (K). These results provide direct experimental constraints on the high-pressure phase stability and structural phase transition of brucite and offer an important reference for understanding the behavior of other hydroxide minerals under extreme conditions.

## 1. Introduction

Brucite [Mg(OH)_2_] is belonging to a typical layered hydroxide mineral with the crystalline structure of hexagonal close packing, which outcrops in the regions of crust and upper mantle and commonly coexists with other magnesium-bearing silicate minerals (i.e., lizardite, antigorite, hydrothermal spinel and chrysotile) [1,2,3,4]. As usual, brucite is formed through the hydration of olivine-rich mantle rocks and is regarded as a byproduct of the olivine serpentinization [5,6]. At ambient conditions, brucite crystallizes in a hexagonal CdI_2_-type structure (space group $P\bar{3}m1$), characterized by layers of edge-sharing MgO_6_ octahedra stacked along the *c* axis orientation. Each oxygen atom is bonded to a hydrogen atom oriented along the threefold axis, forming hydrogen bonds (O–H) that link adjacent octahedral layers. Owing to its unique crystalline structure and chemical composition, the physical and chemical properties of brucite have been extensively investigated in order to explore the volatile transport and migration of the deep Earth mantle. 

Numerous high-pressure investigations have been concentrated on the phase stability and structural phase transition of brucite, which clearly revealed the occurrence of second-ordered structural phase transition from the hydrogen-ordered ($P\bar{3}m1$ symmetry) to hydrogen-disordered ($P\bar{3}$ symmetry) structure under high pressure [7,8,9,10,11,12]. Nevertheless, substantial discrepancies remain regarding the transition pressure of this hydrogen-disordering structural phase transition in brucite. For instance, Catti et al. investigated the phase transition of brucite by powder neutron diffraction without pressure-transmitting medium up to 11 GPa at room temperature [7]. Their results confirmed that brucite endures a second-order phase transition at 6–7 GPa, which is evidenced by the onset of hydrogen disorder and a jump in the *c*/*a* ratio. Contemporaneity, the in situ Raman scattering experiment with the neon as pressure-transmitting medium was performed by Duffy et al. over the pressure range of 0–19.7 GPa at atmospheric temperature [8]. They found that the structural phase transition in brucite occurs at 4.4 GPa, which is revealed by the emergence of several new Raman lines. However, subsequent synchrotron X-ray diffraction results on the natural brucite without pressure-transmitting medium detected no structural phase transition up to 29 GPa [10]. Furthermore, a significantly lower transition pressure of 0.5 GPa was also predicted by Pillai et al. according to the first-principles calculations (DFT) results, accompanied by an evident volume compression of 1.32% [11]. These discrepancies highlight that the structural phase transition pressure of brucite remains poorly constrained. In addition, the pressure-induced discontinuity in electrical conductivity has been proved to be an effective indicator of the structural phase transitions in many minerals [13,14,15]. Therefore, the combination of in situ electrical conductivity and Raman spectroscopy results is essential for better constraining the phase stability and structural phase transition in brucite under high pressure condition.

Temperature is also another one critical factor in influencing the phase stability and structural phase transitions of hydrous minerals [16,17,18]. Previous high-temperature and high-pressure studies on brucite have mainly concentrated on its dehydration behavior and electric conduction mechanism [19,20,21]. As reported by Fuji-ta et al., the electrical conductivity increases sharply near the dehydration boundary, which indicates a mixed electronic–ionic conduction mechanism in brucite [19]. More recently, Guo et al. determined that the brucite completely dehydrates at 950 K and 1300 K under the limited pressure of 3.0 GPa in both open and closed systems, respectively [21]. As we known, the experimental results on the electrical conductivity of hydrous- and carbon-bearing minerals at high temperatures and high pressures have been widely employed in order to not only play a constraint on the structural phase transitions of minerals, but also establish their *P*–*T* phase diagrams [13,14,16,18,22,23,24,25,26,27]. Until now, the correlation between the hydrogen-disordering transition temperature and pressure in brucite, as well as the corresponding *P*–*T* phase diagram, has not been reported. Hence, systematic high-temperature and high-pressure electrical conductivity measurements of brucite across a broad range of pressures and temperatures are crucial for understanding its phase stability and structural phase transition under extreme conditions.

In the present study, in situ Raman scattering and *AC* complex impedance spectroscopy measurements were conducted on brucite in a diamond anvil cell (DAC) to explore its hydrogen-disordering transformation under different hydrostatic environments over the temperature range of 298–873 K and pressures up to 20.2 GPa. The transition temperature of brucite was precisely determined at three representative pressure points (e.g., 1.8, 3.2, 4.5 GPa). On the basis of high-temperature and high-pressure electrical conductivity results, a comprehensive phase diagram of brucite was successfully constructed.

## 2. Experimental Procedure

### 2.1. Sample Preparation

In this study, the natural brucite crystal were obtained from the Kuandian region, Liaoning Province, China. The initial sample was polished and crushed into the fine powder using an agate mortar. The crystallographic structure of the pristine sample was characterized by the wavelength of 0.7107 Å in the Mo Kα radiation of Rigaku SmartLab X-ray diffractometer (Rigaku Corporation, Tokyo, Japan), which is installed at the Key Laboratory of High-Temperature and High-Pressure Study of the Earth’s Interior, Institute of Geochemistry, Chinese Academy of Sciences. The measurements were carried out in Bragg–Brentano reflection geometry at 40 kV and 20 mA over an angular range (2*θ*) range of 6–36°.

### 2.2. XRD Investigation

**Figure 1:**
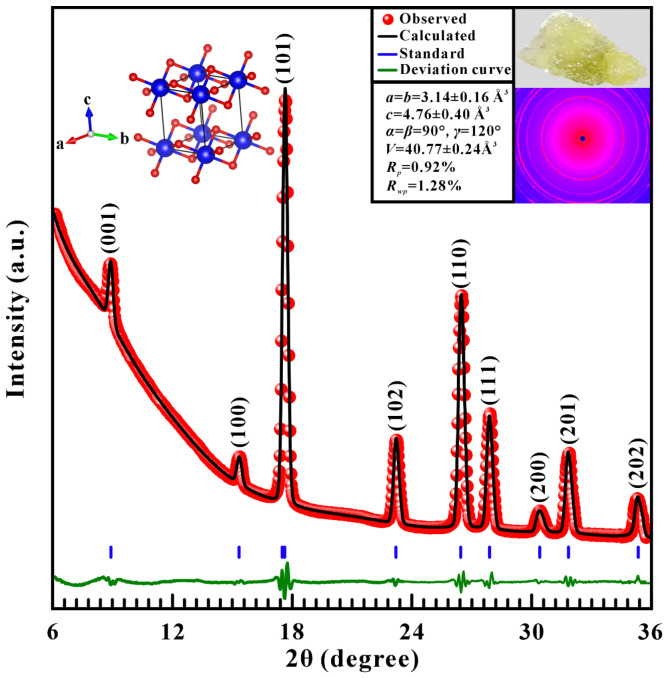
The structural refinement and powder XRD pattern of brucite under ambient conditions. The red solid circles and its corresponding black curve stand for the Rietveld fittings for the observed and calculated results, respectively. Blue vertical bars represent the standardized positions of Bragg peaks. The green solid line represents the deviation curve between the calculated and observed data. Each diffraction peak is labeled with its corresponding Miller indices. Inset: The optical microscope image and the refined lattice parameters of the original brucite sample.

The obtained diffraction patterns were analyzed by Rietveld refinement using the GSAS-II software (V5831) package. As shown in Figure 1, all diffraction peaks can be clearly indexed to a hexagonal lattice with space group $P\bar{3}m1$, corresponding to the brucite-type (CdI_2_) layered structure. The refined unit-cell parameters are *a* = *b* = 3.14 ± 0.16 Å, *c* = 4.76 ± 0.40 Å, *α* = *β* = 90°, *γ* = 120° and *V* = 40.77 ± 0.24 Å^3^, respectively, which are in agreement with previously reported values [28,29]. An elevated background is observed at low 2θ angles (<20°), which is mainly attributed to air scattering and diffuse scattering from the sample environment. Such phenomena are commonly enhanced in the low-angle region in reflection geometry and do not affect the peak positions or relative intensities used for structural refinement [30,31,32,33] As shown in Figure S1, the phase purity of sample has been further confirmed by the Cu Kα XRD measurements over a wide angular range of 6–120°, which shows excellent agreement with the standard reference pattern (ICDD PDF No. 18-0802).

### 2.3. High-Pressure Raman Spectroscopy Measurements

High-pressure Raman scattering experiments on brucite were conducted up to 20.2 GPa using a symmetric diamond anvil cell (DAC) (Shanghai Olordia Superhard Materials Co., Ltd., Shanghai, China) equipped with anvils of 200 μm culet diameter and an 8.5° bevel angle. A sheet of T-301 stainless steel gasket was pre-indented to a thickness of ~50 µm at around 10.0 GPa; then, a 100 µm diameter hole was drilled at the central part to serve as the sample chamber using a laser drilling machine (Beijing Shentie Optoelectronic Equipment Co., Ltd., Beijing, China). Afterwards, pre-compressed brucite powder pellets and the pressure-transmitting medium were then loaded into the sample chamber along with a small ruby ball (grain size ~5 μm) used for pressure calibration. The applied pressure was calibrated based on the wavenumber shift of the R_1_ fluorescence line of Cr^3+^ in ruby [34]. In this study, the 4:1 methanol–ethanol mixture (ME) was used as the pressure-transmitting medium to achieve hydrostatic condition in the sample chamber of DAC, whereas no pressure-transmitting medium (NPT) was utilized for the non-hydrostatic condition. The high-pressure Raman scattering measurements of brucite were performed using the Renishaw Invia Raman microscope (Renishaw plc, Gloucestershire, UK) in the backscattering configuration equipped with a 514.5 nm Ar laser. The acquisition time and laser power were set to 120 s and 50 mW, respectively. Raman spectra were collected over the wavenumber ranges of 200–800 cm^−1^ and 3200–3800 cm^−1^, which correspond to the lattice vibrational and hydroxyl stretching modes of brucite, respectively. Furthermore, each acquired spectrum was processed by the baseline calibration; then, the positions of the Raman peaks were precisely extracted using the PeakFit software (V4.12).

### 2.4. High-Temperature and High-Pressure Electrical Conductivity Measurements

Four column-type DAC with the culet diameter of 300 μm and bevel angle of 10° was utilized for high-temperature and high-pressure electrical conductivity measurements over the wide temperature range of 298–873 K and pressure range of 0.5–19.6 GPa. The gasket of T-301 stainless steel was still pre-indented to ~10.0 GPa; then, the 250 μm diameter hole was drilled at the center of pre-indented area. Subsequently, the mixture of boron nitride and epoxy powder was compacted into the sample chamber up to ~15.0 GPa so as to insulate the electrodes from the metallic gasket; then, another new central hole with a diameter of 150 μm was drilled to serve as the insulating sample chamber. The remaining surface of gasket was coated with insulating cement to ensure complete electrical insulation between two symmetric electrode leads and the metallic gasket. Two slices of platinum electrodes were symmetrically placed on the upper and lower counterparts of sample chamber. Meantime, high temperature was generated by double external electrical resistance heating furnaces tightly wrapped around the tungsten carbide base. The temperature was monitored by a *k*-type thermocouple (Jiangsu Hongguang Electric Heating Appliance Co., Ltd., Yancheng, China) adhered to the side face of diamond anvil. To avoid the introduction of additional impurities and to ensure good contact between electrodes and sample, no pressure-transmitting medium was applied during the measurements. The electrochemical impedance spectroscopy (EIS) of brucite was collected using a Solartron-1260 impedance spectroscopy analyzer (Solartron Analytical, Farnborough, UK) over a wide frequency range of 10^−1^–10^7^ Hz. The measurement principles and experimental procedures are described in our previous work in detail [35].

## 3. Results and Discussion

### 3.1. Raman Spectroscopy Results at High Pressure and Atmospheric Temperature

**Figure 2:**
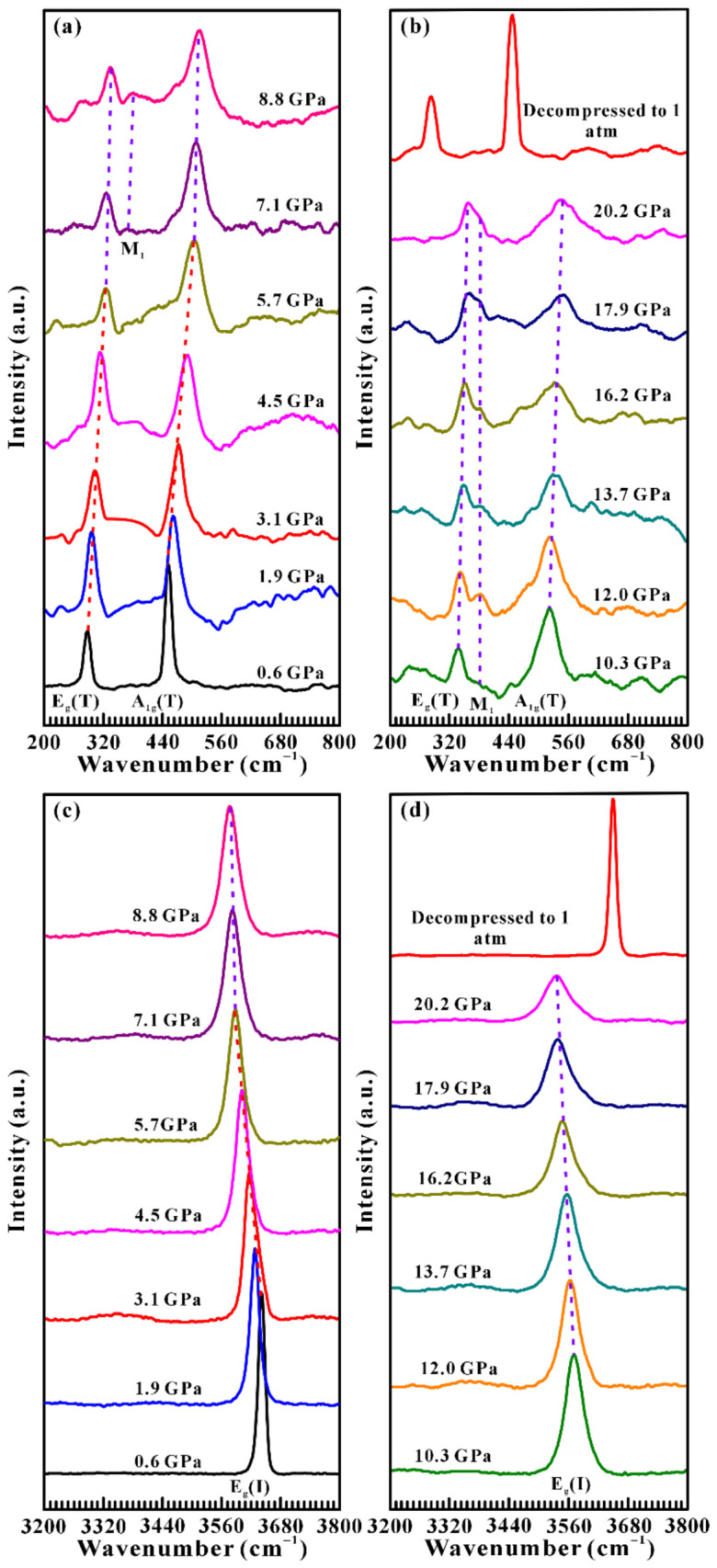
Representative Raman spectra of brucite in the pressure range of 0.6–20.2 GPa and the Raman spectrum of the recovered sample after released to 1 atm under non-hydrostatic condition. (**a**,**b**) the lattice vibrational mode (200–800 cm^−1^); (**c**,**d**) the hydroxyl stretching mode (3200–3800 cm^−1^).

The vibrational properties of brucite were investigated via Raman spectroscopy up to 20.2 GPa at atmospheric temperature. Figure 2 presents the representative Raman spectra of brucite as a function of pressure within the wavenumber range of 200–800 cm^−1^ and 3200–3800 cm^−1^ under non-hydrostatic condition. As shown in Figure 2a, two prominent Raman peaks at the positions of 284.5 and 451.1 cm^−1^ were identified at 0.6 GPa, which correspond to the E_g_ (T) and A_1g_ (T) modes of brucite, respectively [2,8]. In the meantime, a distinct hydroxyl stretching peak at the position of 3642.5 cm^−1^ was detected within the wavenumber range of 3200–3800 cm^−1^ (Figure 2c), which can be assigned to the E_g_ (I) mode of brucite. Here, the T and I stand for the translation mode and internal mode, respectively. Overall, the Raman peaks acquired at 0.6 GPa are in excellent agreement with previous investigations [3,8].

At 0.6–5.7 GPa, the A_1g_ (T) and E_g_ (T) Raman modes of sample uniformly shifts towards higher wavenumbers with increasing pressure and exhibit an apparent blueshift characterization, which reflects a substantial decrease in the unit cell volume. On the contrary, the E_g_ (I) mode tends to shift towards lower wavenumbers and displays a characteristic redshift behavior. This behavior is related to the subtle changes in the local electric field and interlayer interactions upon compression [36]. Notably, at the critical pressure of 5.7 GPa, several extremely weak new Raman signals emerged on the right side of E_g_ (T) mode, which may imply the occurrence of structural phase transition in brucite. However, a detailed investigation for the newly emerged features at 5.7 GPa was hindered by the low signal-to-noise ratio during the measurements. Upon further compression beyond 5.7 GPa, the intensity of these signals gradually increased with the rise of pressure, and later, a distinct Raman peak at approximately 371.4 cm^−1^ can be clearly identified at 7.1 GPa, which can be denoted as M_1_ mode. A similar phenomenon was also observed in the previous Raman scattering experiments performed by Duffy et al., and they reported that the M_1_ peak first appeared at 14.3 GPa under non-hydrostatic condition [8]. In the pressure range of 5.7–20.2 GPa, three Raman peaks including A_1g_ (T), M_1_ and E_g_ (T) still move towards higher wavenumbers, accompanied by the progressive sharpening of the M_1_ mode and broadening of A_1g_ (T) and E_g_ (T) modes. Meantime, the E_g_ (I) mode continues to shift towards lower wavenumbers with a significant reduction in peak intensity. The observed band broadening is attributed to increasing hydrogen disorder under compression, which leads to a range of O–H bond distances, orientations, and strengths [37]. Upon decompression to ambient conditions, the Raman spectrum completely reverts to its original state, indicating that the pressure-induced structural phase transition in brucite is reversible under non-hydrostatic conditions.

**Figure 3:**
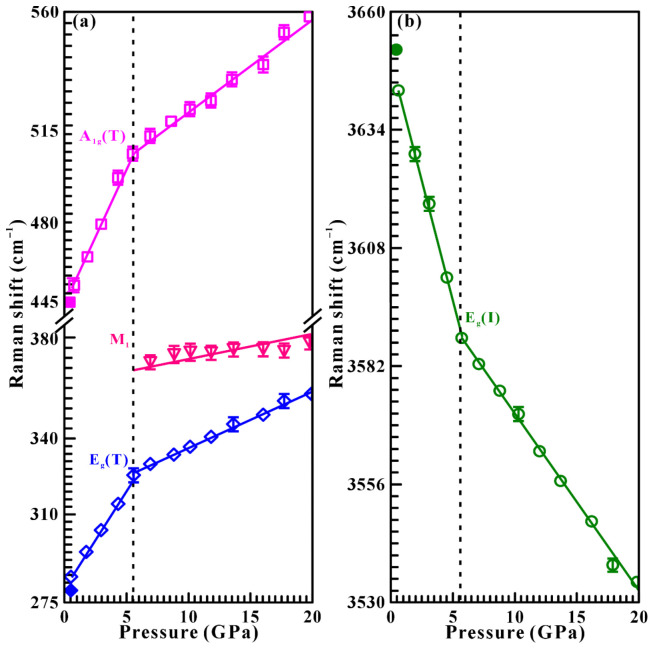
Pressure-dependent Raman shifts of brucite in the pressure range of 0.6–20.2 GPa under non-hydrostatic condition. (**a**) The lattice vibrational mode (200–800 cm^−1^); (**b**) the hydroxyl stretching mode (3200–3800 cm^−1^). Here, open symbols represent the compression data, and filled symbols represent decompression data.

Moreover, the pressure dependence of Raman shifts and their corresponding fitting results (∂*ω*/∂*P*, cm^−1^GPa^−1^) are presented in detail in Figure 3 and Table 1 to further explore the occurrence of the structural phase transition in brucite. Below 5.7 GPa, the Raman modes of E_g_ (T) and A_1g_ (T) exhibit some typical blueshift behaviors with steep and positive slopes of 7.74 and 10.62 cm^−1^GPa^−1^, respectively, whereas the E_g_ (I) mode shows obvious redshift with a steep and negative slope of −10.59 cm^−1^GPa^−1^. Above 5.7 GPa, all Raman modes (E_g_ (T), M_1_, A_1g_ (T) and E_g_ (I)) shift with gentler rates of 2.24, 1.20, 3.67 and −3.90 cm^−1^GPa^−1^, respectively. The discontinuous pressure-dependent Raman shifts for brucite observed at 5.7 GPa clearly divided the compression process into two distinct pressure ranges, providing strong evidence for the occurrence of phase transition in brucite under non-hydrostatic condition.

**Figure 4:**
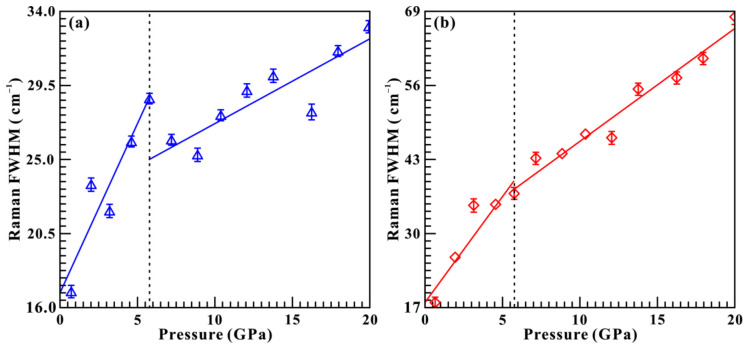
The evolution of Raman FWHMs as a function of pressure for the characteristic Raman modes of (**a**) E_g_ (T) and (**b**) E_g_ (I) under non-hydrostatic conditions.

In addition, we also analyzed the Raman full width at half-maximum (FWHM) of sample under non-hydrostatic condition. Figure 4 shows the pressure-dependent Raman FWHMs (∂*F*/∂*P*, cm^−1^GPa^−1^) of E_g_ (T) and E_g_ (I) modes, and their corresponding fitting results are listed in Table 2 in detail. It made clear that the Raman FWHMs of E_g_ (T) and E_g_ (I) modes increased with the enhancement of pressure at relatively steep rates of 2.04 and 3.71 cm^−1^GPa^−1^ in the pressure range of 0.6–5.7 GPa, respectively. When the pressure exceeded 5.7 GPa, a marked drop in the slope was observed, which increased at relatively gentler rates of 0.51 and 1.98 cm^−1^GPa^−1^. Our observations revealed that both of E_g_ (T) and E_g_ (I) modes show evident discontinuities on the pressure dependence of Raman FWHMs at 5.7 GPa. To provide further insight into the pressure-induced hydrogen-disordering transformation of brucite, the evolution of Raman peak intensities of the dominant E_g_ (I) mode was analyzed in detail. As shown in Figure S2a, the intensity of E_g_ (I) mode decreases monotonically with increasing pressure, whereas a clear discontinuity can be identified at 5.7 GPa. Notably, this discontinuity in the pressure-dependent Raman peak intensity is consistent with the corresponding variations in Raman FWHMs. In sum, these results confirm that brucite underwent a structural phase transition at 5.7 GPa under non-hydrostatic conditions.

For the hydrostatic condition, the high-pressure Raman spectra of brucite were collected up to 19.5 GPa at room temperature using ME as the pressure-transmitting medium. Similarly, high-pressure Raman spectra, pressure-dependent Raman shifts, Raman full width at half-maxima, together with their corresponding fitting results over the pressure range of 0.8–19.5 GPa were displayed in detail in the Supporting Information (Figures S3–S5 and Tables S1 and S2). Upon compression, the structural phase transition of brucite was detected at 3.6 GPa under hydrostatic condition, which was evidenced by the emergence of new Raman features in conjunction with the discontinuities in pressure dependence of Raman shifts and FWHM results. Compared with the transition pressure of 5.7 GPa under the non-hydrostatic condition, a lower phase transition pressure of 3.6 GPa was observed under the hydrostatic condition. Therefore, it is clear that the structural transition pressure from the ordered ($P\bar{3}m1$ symmetry)-to-disordered ($P\bar{3}$ symmetry) brucite was promoted by 2.1 GPa under hydrostatic condition, which can be ascribed to the influence of deviatoric stress within the DAC sample chamber. An analogous phenomenon has also been detected in other alkali-earth metal ionic hydroxide, such as Ca(OH)_2_, which crystallizes in same hexagonal structure and $P\bar{3}m1\;$space group [38]. As the pressure released to atmospheric pressure, the Raman spectrum also recovered to its initial state, further demonstrating the reversibility of the hydrogen-disordering transformation in brucite under different hydrostatic environments.

### 3.2. Electrical Conductivity Results at High Pressure and Atmospheric Temperature

**Figure 5:**
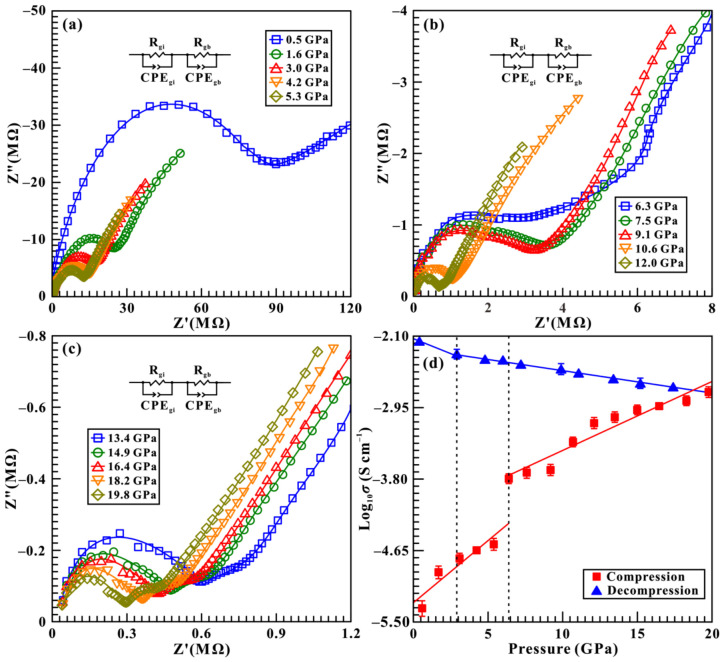
(**a**–**c**) Cole–Cole diagram of impedance spectra for brucite within the pressure range of 0.5–19.8 GPa. Z′ and Z″ represent the real and imaginary parts of the complex impedance, respectively. All these colored curves represent the fitting results of impedance spectra on brucite. (**d**) Pressure-dependent electrical conductivity of brucite upon compression and decompression. In this figure, the solid and dashed lines serve as guides to the eye.

The Cole–Cole plots of the impedance spectra for brucite in the pressure range of 0.5–19.8 GPa and atmospheric temperature are illustrated in Figure 5a–c, which were measured within the frequency range of 10^−1^–10^7^ Hz under a sinusoidal voltage of 1.0 V. Obviously, the acquired impedance spectra consist of a semicircular arc in the high-frequency region (10^2^–10^7^ Hz) and an oblique tail in the low-frequency region (10^−1^–10^2^ Hz), which correspond to the grain-interior resistance (*R*_gi_) and grain-boundary resistance (*R*_gb_) of brucite sample, respectively. To accurately determine the resistance of brucite, all of these collected impedance spectra were fitted using an appropriate equivalent circuit with the ZView software (V3.5). The equivalent circuit comprise of a resistor (R) connected in parallel with a constant-phase element (CPE) [13,39,40,41]. Then, the electrical conductivity (*σ*) of brucite was calculated:

(1) σ = L/SR

where the *L* and *S* represent the sample thickness (cm) and the cross-sectional area of electrodes (cm^2^), respectively. The logarithmic electrical conductivity of brucite as a function of pressure upon pressurization and depressurization is presented in Figure 5d. Upon compression, the electrical conductivity of sample progressively increases with the rise of pressure, and followed by two distinct pressure regions can be clearly identified: (i) in the pressure range of 0.5–5.3 GPa, the electrical conductivity increase with a relatively steep slope of 0.15 S cm^−1^GPa^−1^; and (ii) from 6.3 GPa to 19.6 GPa, the electrical conductivity continues to increase but at a relatively gentler rate of 0.08 S cm^−1^GPa^−1^. In light of the abrupt variation in slope, an inflection point in electrical conductivity was identified at 6.3 GPa, which provides robust evidence for the occurrence of structural phase transition in brucite. The transition pressure of 6.3 GPa derived from high-pressure electrical conductivity measurements is consistent with the 5.7 GPa acquired from Raman spectroscopy under the non-hydrostatic condition. Upon decompression, the electrical conductivity of sample exhibited tiny variation across the pressure range of 19.8–2.9 GPa. Upon further decompressed to ambient conditions, a marked decrease in the logarithm of electrical conductivity from −2.34 to −2.15 S cm^−1^ was observed, which indicates the reversibility of the structural phase transition in brucite. This observation is consistent with our high-pressure Raman spectroscopy results under different hydrostatic environments.

### 3.3. Transformation Pressure of the Pressure-Induced Hydrogen-Disordering in Brucite

In the present work, we determined a pressure-induced structural phase transition in brucite at ~5.7 GPa, as evidenced by the appearance of new Raman features and the discontinuities observed in the pressure-dependent Raman shifts, FWHMs and electrical conductivity under the non-hydrostatic condition. Meantime, under the hydrostatic condition, this transformation of brucite was observed at 3.6 GPa using ME as the pressure-transmitting medium. It is well known that deviatoric stress can significantly influence the high-pressure physical properties of some minerals and materials [35,40]. In fact, previous studies on the phase stability of brucite under high pressure have reported inconsistent results. A detailed comparison between our acquired results and previously reported results was made and summarized in Table 3. Under the hydrostatic condition, the transition pressure with ME as the pressure-transmitting medium is slightly lower than those reported by Duffy et al. and Zhu et al., who employed neon and liquid argon as the pressure-transmitting media, respectively; the results are however slightly higher than those derived by Shinoda et al. using fluorocarbon fluid as the pressure-transmitting medium [8,9,12]. These minor discrepancies in the reported transformation pressure of brucite likely stem from the choice of PTM. Under the non-hydrostatic condition, our acquired transition pressure is comparable to that reported by Catti et al. via powder neutron diffraction experiments [7]. However, our result of 5.7 GPa differs significantly from those of Ma et al. and Pillai et al., who did not detect any phase transition and predicted a much lower transition pressure of 0.5 GPa with NPT [10,11]. Therefore, the discrepancies in the structural phase transition of brucite probably cannot be solely attributed to the influence of deviatoric stress or the type of pressure-transmitting media, but are also affected by differences in experimental methods and data resolution. All of those above-mentioned factors jointly control the occurrence of structural phase transition in brucite. In addition, in comprehensive considerations of Raman spectroscopy, electrical transport property observation, the hydrogen-disordering transformation in brucite is confirmed to be reversible under different hydrostatic environments.

## 4. The High-*T* and High-*P* Phase Diagram of Brucite

**Figure 6:**
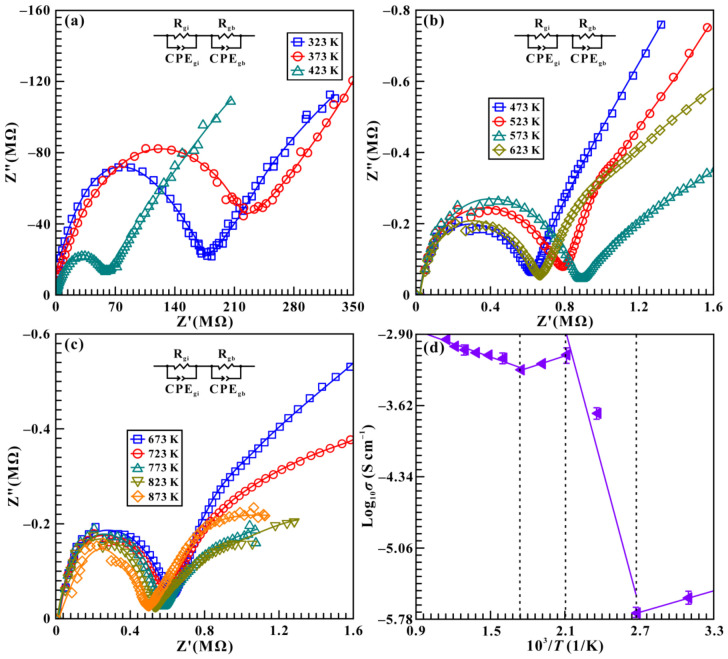
Cole–Cole plots of impedance spectra for brucite measured at a given pressure of 4.5 GPa over the temperature range of 323–873 K, as well as the corresponding logarithmic electrical conductivity as a function of reciprocal temperature. (**a**) 323–423 K; (**b**) 473–623 K; (**c**) 673–873 K. The colored curves represent the fitting results of impedance spectra on brucite; (**d**) the logarithmic electrical conductivity of sample as a function of reciprocal temperature. Solid and dashed lines are provided as visual guides.

As we know, temperature plays a vital role in influencing the phase transition pressure of hydrous minerals, which has been well demonstrated in our previous studies [13,16,17,24]. In order to explore the effect of temperature on the hydrogen-disordering transformation in brucite and to further construct its high-*T* and high-*P* phase diagram, a series of electrical conductivity measurements were conducted at three fixed pressures (1.8, 3.2, and 4.5 GPa) over the temperature range of 323–873 K, as shown in Figure 6, Figures S4 and S5. Figure 6a–c display the Cole–Cole plots of impedance spectra for brucite up to 873 K with a temperature interval of 50 K at 4.5 GPa. Simultaneously, the corresponding logarithmic electrical conductivity as a function of reciprocal temperature is presented in Figure 6d. As temperature increases from 323 to 373 K, the electrical conductivity decreases from −2.34 to −2.15 S cm^−1^, which is likely attributed to the removal of evaporable water from the initial brucite sample [42]. Within the respective temperature ranges of 373–473 K and 573–873 K, the electrical conductivity of sample increases with rising temperature, which exhibits a clear positive correlation between temperature and electrical conductivity. By contrast, the electrical conductivity of brucite decreases with the rise of temperature at 473–573 K and demonstrates a characteristic negative temperature-dependent electrical conductivity. Overall, this pronounced reduction in electrical conductivity observed at 473 K under the given pressure of 4.5 GPa is probably caused by the structural phase transition of brucite from $P\bar{3}m1$ to$\;P\bar{3}$ symmetry, which initiates at 473 K and completes at 573 K. To further evaluate the relationship of temperature and structural phase transition pressure in brucite, additional high-temperature and high-pressure electrical conductivity measurements for brucite were conducted at 1.8 and 3.2 GPa. Similar to the observations obtained at 4.5 GPa, the corresponding structural phase transition temperatures of brucite were determined to be 573 and 823 K at fixed pressures of 1.8 and 3.2 GPa, respectively (Figures S6 and S7). In comparison with the high-temperature and high-pressure results acquired at 4.5 GPa, it is evident that the transition temperature of the hydrogen-disordering transformation in brucite decreases with increasing pressure, showing a characteristic negative pressure dependence on temperature.

**Figure 7:**
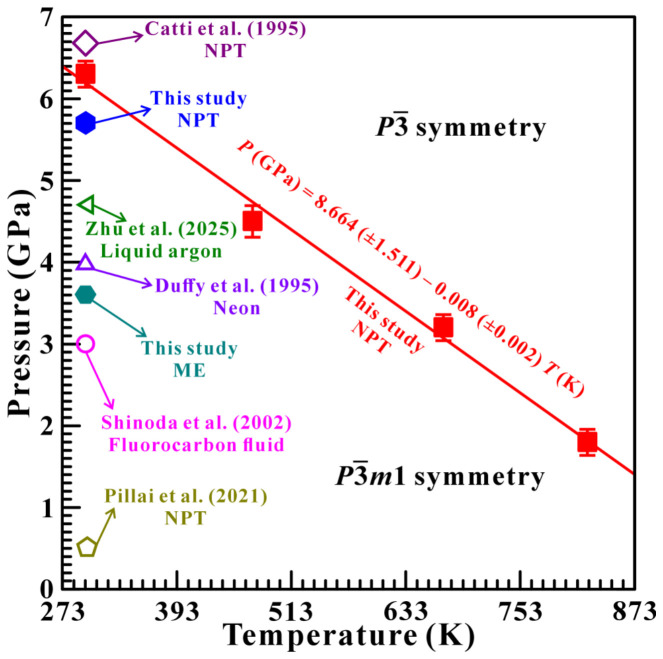
The proposed *P*–*T* phase diagram of brucite investigated under conditions of temperatures up to 873 K and pressures up to 7.0 GPa. The red solid line is the phase boundary from $P\bar{3}m1$ to$\;P\bar{3}$ symmetry in brucite. In here, the solid and hollow symbols are used to distinguish our work from previous work [7,8,9,11,12].

Furthermore, based on these acquired high-*T* and high-*P* electrical conductivity results, the *P*–*T* phase diagram of brucite over the temperature range of 298–873 K and pressures up to 7.0 GPa was established, as shown in Figure 7 in detail. According to this phase diagram, the phase boundary of the structural transition from $P\bar{3}m1$ to$\;P\bar{3}$ symmetry in brucite can be described as

(2) P (GPa)=8.664 (±1.511) − 0.008 (±0.002) T (K)

where *P* and *T* denote the experimental pressure (GPa) and temperature (K), respectively. Noteworthily, it is the first time that the high *P*–*T* phase diagram of hydrogen-disordering transformation in brucite was constrained. In addition, the Clausius–Clapeyron (CCR) relation is widely applied to quantify the pressure–temperature dependence of a phase transition or mineral reaction [43,44,45],

d*T*/d*P* = ∆V/∆S (3)

where d*T*/d*P*, ∆V and ∆S denote the Clapeyron slope, molar volume change and entropy change, respectively. As pointed out by Pillai et al., the occurrence of the structural phase transition in brucite is accompanied by a small volume compression of approximately 1.32%, which indicates a negative value for ΔV [11]. Moreover, Duffy et al. conducted high-pressure Raman scattering experiments of brucite and revealed a reduction in the symmetry of unit cell due to the occurrence of the structural phase transition; thus, the ΔS is a positive value [8]. Consequently, the Clapeyron slope (d*T*/d*P*) for the hydrogen-disordering transformation boundary of brucite is negative, which is consistent with our above-acquired electrical conductivity results at high temperature and high pressure (−0.008 GPaK^−1^).

## 5. Conclusions

In this work, a series of in situ Raman spectroscopy and electrical conductivity measurements were carried out to investigate the phase stability and structural phase transition of brucite under different hydrostatic environments at temperatures of 298–873 K and pressures of 0.5–20.2 GPa in a DAC. The emergence of new Raman features, discontinuities in the pressure-dependent Raman shifts, FWHMs of E_g_ (T) and E_g_ (I) modes along with electrical conductivity collectively indicated a pressure-induced structural phase transition of brucite at 5.7 GPa under the non-hydrostatic condition. This was followed by a lower transition pressure of 3.7 GPa, obtained under the hydrostatic condition. This discrepancy is likely related to the influence of deviatoric stress within the sample chamber of DAC. Moreover, the long-standing controversy in the phase transition pressure of brucite was further discussed through detailed comparison with previous experimental and theoretical studies. Furthermore, the high-temperature and high-pressure electrical conductivity measurements conducted at three fixed pressures of 1.8, 3.2, and 4.5 GPa over the temperature range of 298–873 K show that the transition temperature progressively decreases with increasing pressure, revealing a negative dependence of the transition temperature on pressure. Finally, the high *P*–*T* phase diagram for the hydrogen-disordering transformation in brucite was successfully established for the first time, and the phase boundary from $P\bar{3}m1$ to$\;P\bar{3}$ symmetry can be described as P (GPa) = 8.664 (±1.511) − 0.008 (±0.002) T (K). These results not only provide direct experimental constraints on the high-pressure phase stability and structural phase transition of brucite. However, they also offer an important reference for understanding the behavior of other hydroxide minerals under extreme conditions.

## Figures and Tables

**Table 1:** Pressure-dependent Raman shifts (d*ω*/d*P*, cm^−1^GPa^−1^) for brucite under non-hydrostatic condition. Here, *ω* (cm^−1^) and *P* (GPa) represent the Raman wavenumber and pressure, respectively.

Pressure	Mode (cm^−1^)	d*ω*/d*P* (cm^−1^GPa^−1^)
0.6–5.7 GPa	E_g_ (T) (284.5)	7.74
	A_1g_ (T) (451.1)	10.62
	E_g_ (I) (3642.5)	−10.59
5.7–20.2 GPa	E_g_ (T) (324.6)	2.24
	M_1_ (371.4)	1.02
	A_1g_ (T) (503.1)	3.67
	E_g_ (I) (3588.3)	−3.9

**Table 2:** Pressure-dependent Raman FWHMs (d*F*/d*P*, cm^−1^GPa^−1^) for brucite under non-hydrostatic condition. Here, *F* (cm^−1^) and *P* (GPa) represent the Raman FWHM and pressure, respectively.

Pressure	Mode (cm^−1^)	d*F*/d*P* (cm^−1^GPa^−1^)
0.6–5.7 GPa	E_g_ (T) (284.5)	2.04
	E_g_ (I) (3642.5)	3.71
5.7–20.2 GPa	E_g_ (T) (324.6)	0.51
	E_g_ (I) (3588.3)	1.98

**Table 3:** Comparison of the pressure-induced structural phase transition of brucite under different hydrostatic environments and room temperature.

Methods	Pressure Medium	P_tr_	Refs
Raman spectroscopy	NPT	5.7 GPa	This study
	Mixture of methanol and ethanol (4:1 volume ratio)	3.6 GPa	
Electrical conductivity	NPT	6.3 GPa	This study
Raman spectroscopy	Neon	4.4 GPa	[8]
Powder neutron diffraction	NPT	6.0–7.0 GPa	[7]
Infrared synchrotron radiation	Fluorocarbon fluid	3.0 GPa	[9]
Synchrotron X-ray diffraction	NPT	–	[10]
First-principles calculations	NPT	0.5 GPa	[11]
Fourier transform infrared spectra	Liquid argon	4.7 GPa	[12]

## Data Availability

The data that support the findings of this study are available from the corresponding author upon reasonable request.

## Supplementary Materials

The following supporting information can be downloaded at: https://www.mdpi.com/article/10.3390/molecules31101631/s1, Figure S1: The structural refinement and powder XRD pattern of brucite under ambient conditions using the Cu Kα.; Table S1: Pressure-dependent Raman shift (dω/dP, cm^−1^GPa^−1^) for brucite under hydrostatic condition.
