# Supplementary material for: Hydrogen-Disordering Transformation and High-Temperature and High-Pressure Phase Diagram of Brucite: Insights from Raman Spectroscopy and Electrical Conductivity
Source: Molecules. 2026 May 12;31(10):1631. doi: 10.3390/molecules31101631 (PMC13209758; doi:10.3390/molecules31101631)
Supplement: Supplementary file 1 [file molecules-31-01631-s001.zip › molecules-4263555-supplementary.pdf]

Hydrogen disordering transformation and high-temperature and high-pressure phase diagram of brucite: Insights from Raman spectroscopy and electrical conductivity

Mingyu Wu[1,3], Lidong Dai[2*], Haiying Hu[2*] and Chuang Li[1,3]

AFFILIATIONS

[1]Key Laboratory of High-Temperature and High-Pressure Study of the Earth's Interior, Institute of Geochemistry, Chinese Academy of Sciences, Guizhou 550081, China;

[2] School of Physics and Electronic Science, Guizhou Normal University, Guiyang 550025, Guizhou, China

[3]University of Chinese Academy of Sciences, Beijing 100049, China

[*]Authors to whom correspondence should be addressed:

dailidong@gznu.edu.cn and huhaiying@gznu.edu.cn

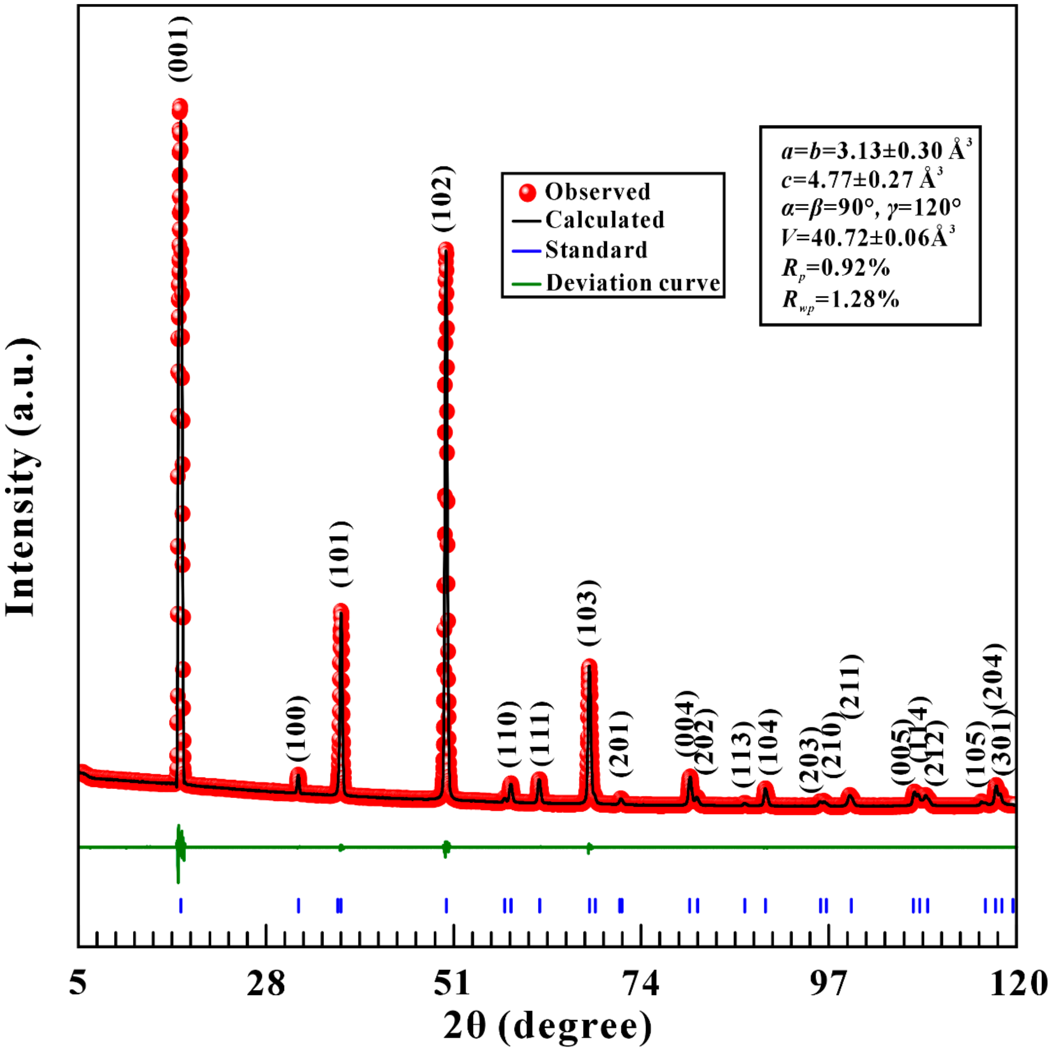

Figure. S1 The structural refinement and powder XRD pattern of brucite under ambient conditions using the Cu Kα. The red solid circles and its corresponding black curve stand for the Rietveld fittings for the observed and calculated results, respectively. Blue vertical bars represent the standardized positions of Bragg peaks. The green solid line is representing the deviation curve between the calculated and observed data. Each diffraction peak is labeled with its corresponding Miller indices. Inset: The refined lattice parameters of sample.

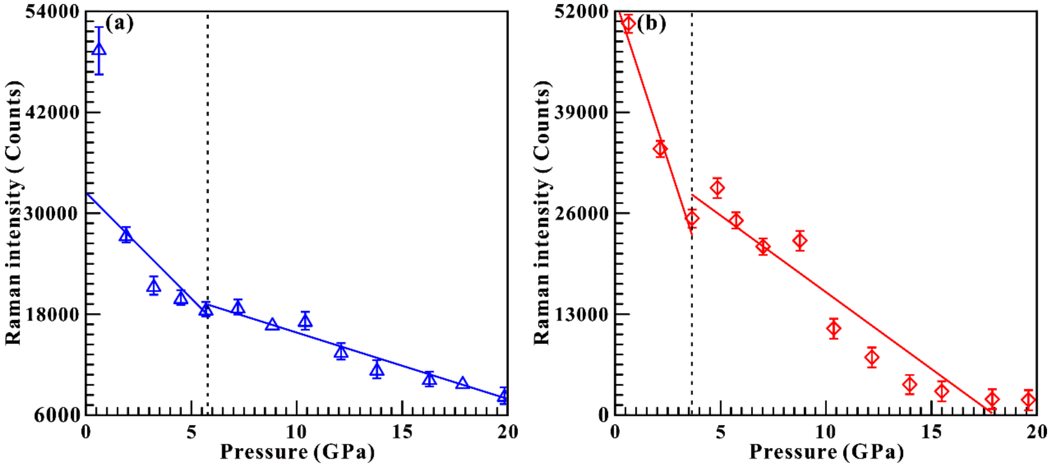

Figure. S2 The evolution of Raman intensities as a function of pressure for the characteristic Raman mode of E_g (I) under (a) non-hydrostatic and (b) hydrostatic conditions.

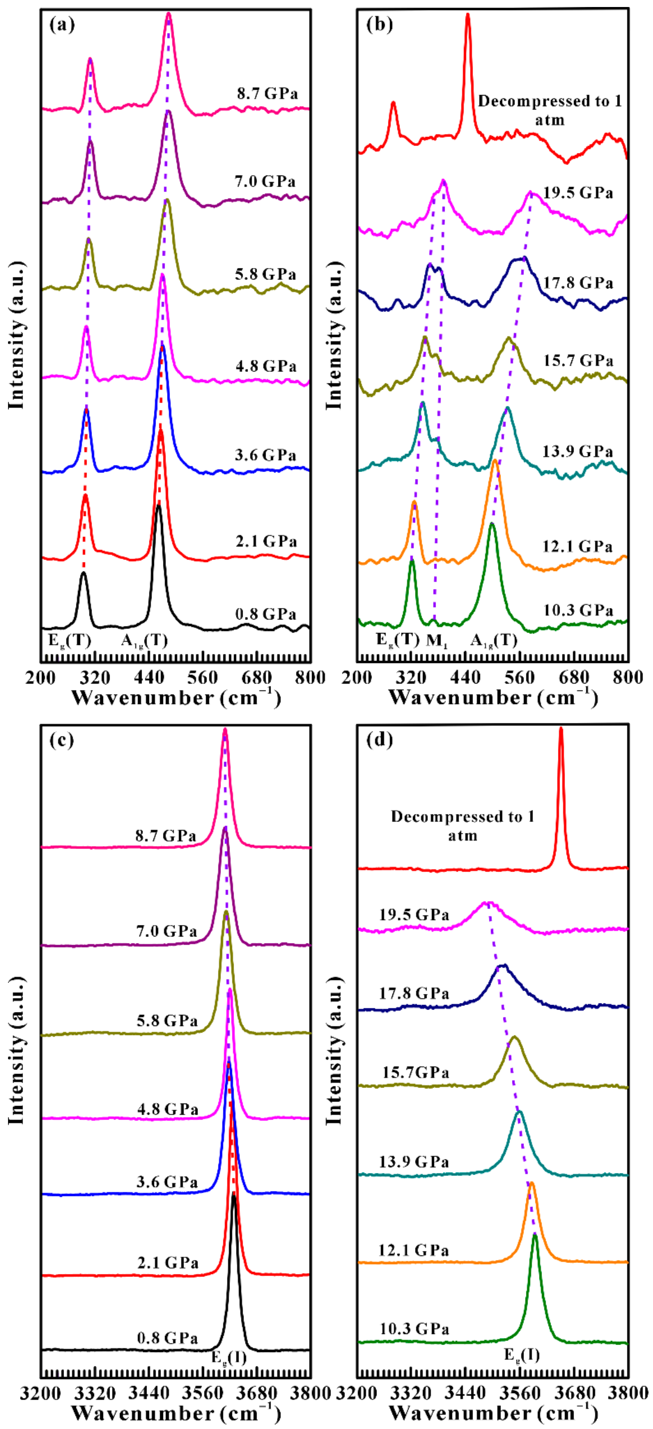

Figure. S3 Representative Raman spectra of brucite in the pressure range of 0.8–19.5 GPa and the Raman spectrum of the recovered sample after release to 1 atm under hydrostatic condition. (a)–(b) the lattice vibrational mode (200–800 cm^{-1}); (c)–(d) the hydroxyl stretching mode (3200–3800 cm^{-1})

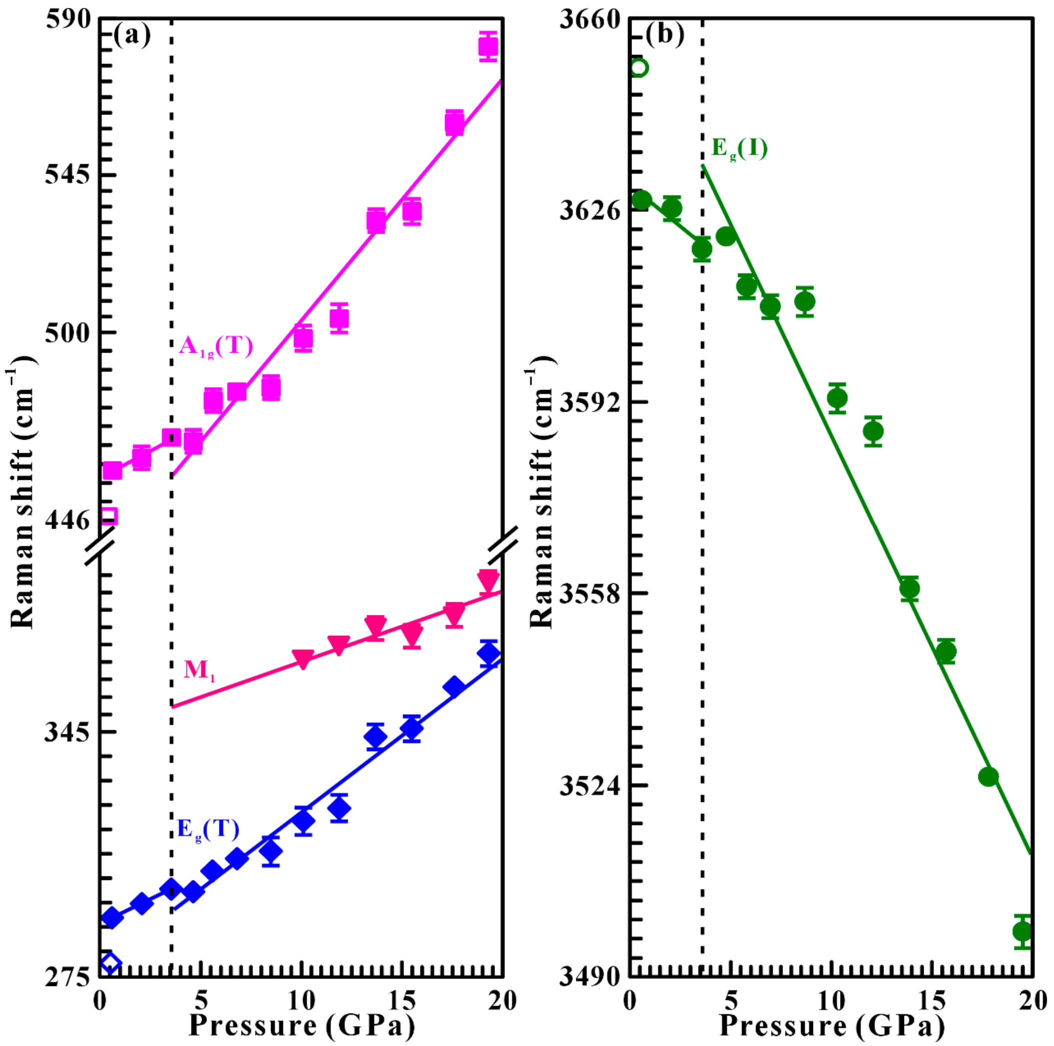

Figure. S4 Pressure-dependent Raman shifts of brucite in the pressure range of 0.8–19.5 GPa under hydrostatic condition. (a) the lattice vibrational mode (200–800 cm^{-1}); (b) the hydroxyl stretching mode (3200–3800 cm^{-1}). Here, filled symbols represent compression data, and open symbols represent decompression data.

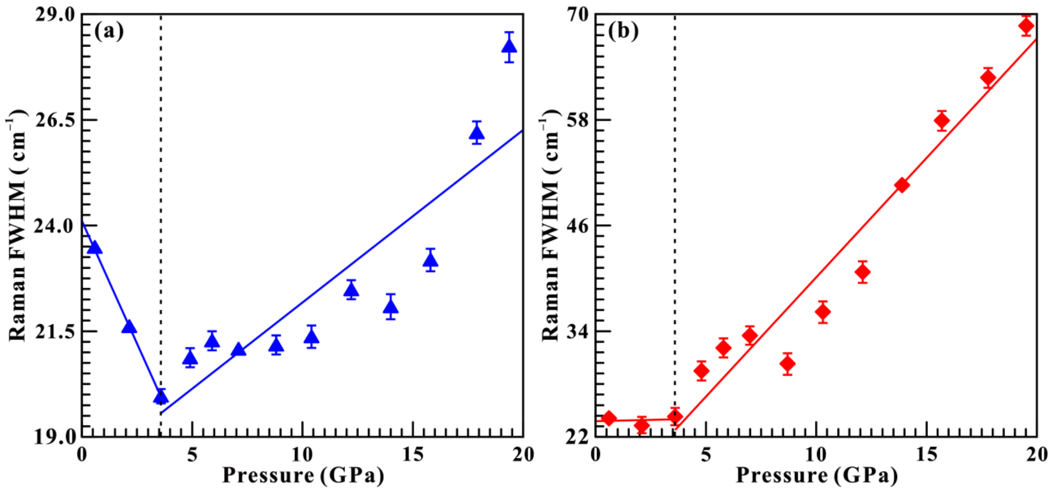

Figure. S5 The evolution of Raman FWHMs as a function of pressure for the characteristic Raman modes of (a) E_g (T) and (b) E_g (I) under hydrostatic condition.

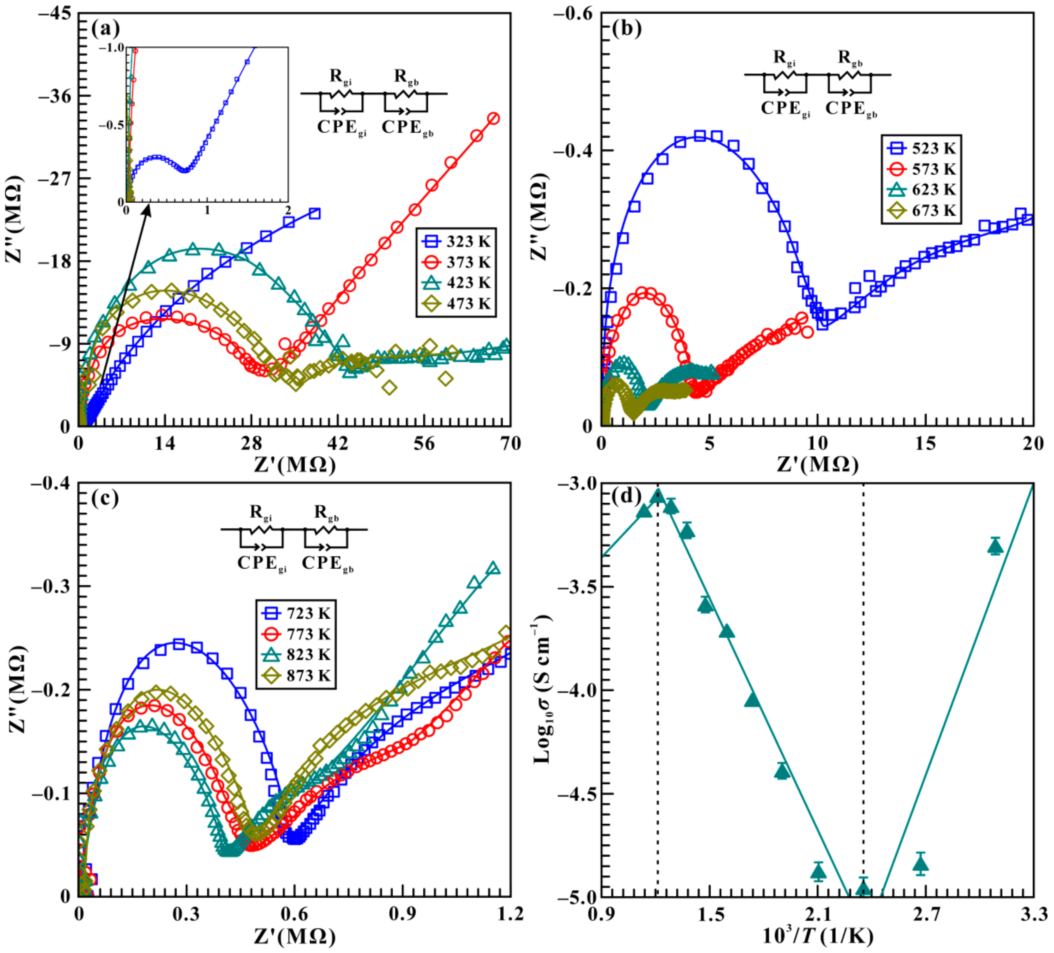

Figure. S6 Cole–Cole plots of impedance spectra for brucite measured at a given pressure of 1.8 GPa over the temperature range of 323–873 K, as well as the corresponding logarithmic electrical conductivity as a function of reciprocal temperature. (a) 323–473 K; (b) 523–673 K; (c) 723–873 K; All these colored curves represent the fitting results of impedance spectra on brucite; (d) the logarithmic electrical conductivity of sample as a function of reciprocal temperature. Solid and dashed lines are provided as visual guides.

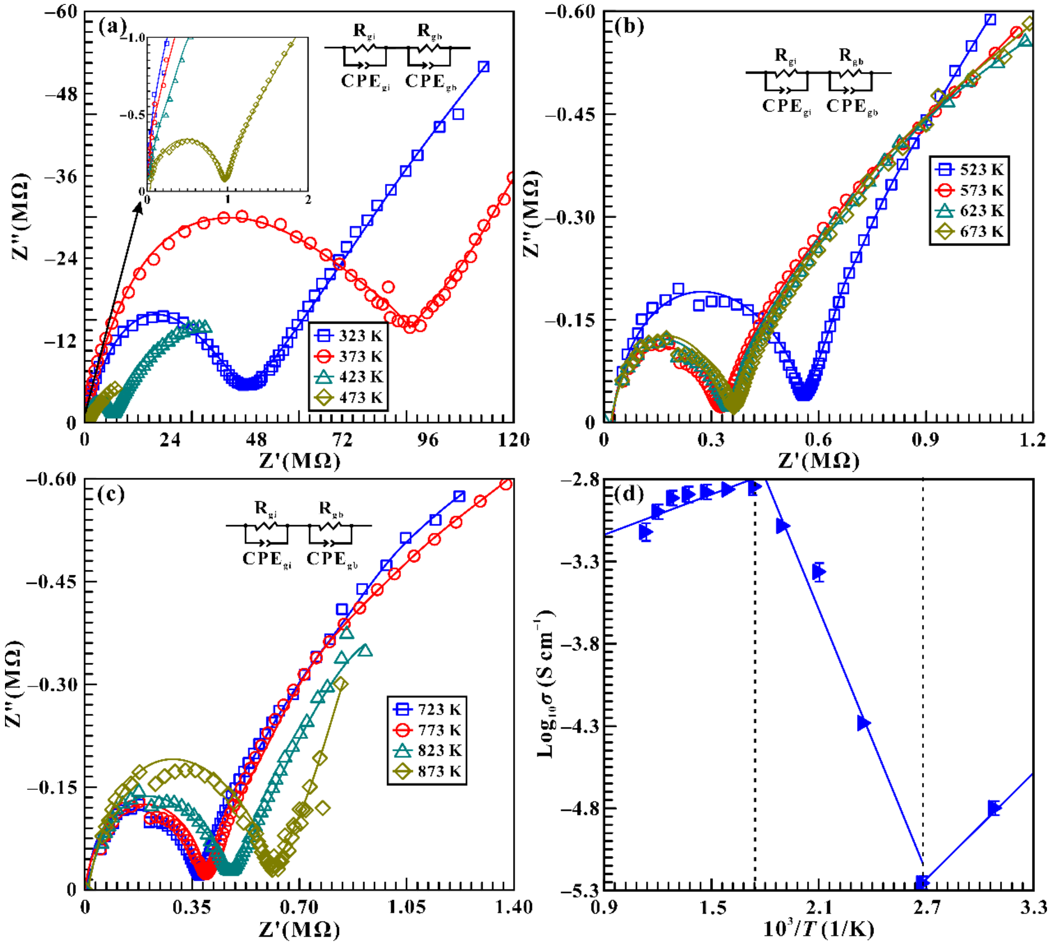

Figure. S7 Cole–Cole plots of impedance spectra for brucite measured at a given pressure of 3.2 GPa over the temperature range of 323–873 K, as well as the corresponding logarithmic electrical conductivity as a function of reciprocal temperature. (a) 323–473 K; (b) 523–673 K; (c) 723–873 K; All these colored curves represent the fitting results of impedance spectra on brucite; (d) the logarithmic electrical conductivity of sample as a function of reciprocal temperature. Solid and dashed lines are provided as visual guides.

Table. S1 Pressure-dependent Raman shift (dω/dP, cm^{-1}GPa^{-1}) for brucite under hydrostatic condition. Here, ω (cm^{-1}) and P (GPa) represent the Raman wavenumber and pressure, respectively.

Pressure	Mode (cm^{-1})	dω/dP (cm^{-1}GPa^{-1})
0.8–3.6 GPa	E_g (T) (291.9)	2.83
	A_{1g} (T) (460.5)	3.13
	E_g (I) (3627.8)	7.98
3.6–19.5 GPa	E_g (T) (300.4)	4.41
	M_1 (366.5)	2.01
	A_{1g} (T) (469.9)	6.90
	E_g (I) (3619.1)	7.47

Table. S2 Pressure-dependent Raman FWHM (dF/dP, cm^{-1}GPa^{-1}) for brucite under hydrostatic condition. Here, F (cm^{-1}) and P (GPa) represent the Raman FWHM and pressure, respectively.

Pressure	Mode (cm^{-1})	dF/dP (cm^{-1}GPa^{-1})
0.8–3.6 GPa	E_g (T) (291.9)	–1.16
	E_g (I) (3627.8)	0.07
3.6–19.5 GPa	E_g (T) (300.4)	0.40
	E_g (I) (3619.1)	2.71
